# Impact of health research on advances in knowledge, research capacity-building and evidence-informed policies: a case study on maternal mortality and morbidity in Brazil: Impacto da pesquisa em saúde nos avanços de conhecimento, construção de capacidades de pesquisa e políticas informadas por evidências: um estudo de caso sobre a morbimortalidade materna no Brasil

**DOI:** 10.1590/1516-3180.2015.01530211

**Published:** 2015-03-17

**Authors:** Antonia Angulo-Tuesta, Leonor Maria Pacheco Santos, Daniel Alves Natalizi

**Affiliations:** 1 PhD. Associate Professor, Faculdade da Ceilândia, Universidade de Brasília, Brasília, DF, Brazil.; 2 PhD. Associate Professor and Head of Department of Public Health, Universidade de Brasília, Brasília, DF, Brazil.; 3 MSc. Postgraduate Student in Human Nutrition Program, Faculdade de Ciências da Saúde, Universidade de Brasília, Brasília, DF, Brazil.

**Keywords:** Health services research, Maternal mortality, Women's health, Maternal health services, Research policy evaluation

## Abstract

**CONTEXT AND OBJECTIVE:**

National health research systems aim to generate high-quality knowledge so as to maintain and promote the population's health. This study aimed to analyze the impact of maternal mortality/morbidity research funded by the Brazilian Ministry of Health and institutional partners, on the dimensions: advancing in knowledge, research capacity-building and informing decision-making, within the framework of the Canadian Academy of Health Sciences.

**DESIGN AND SETTING:**

Descriptive study based on secondary data, conducted at a public university.

**METHODS:**

The advancing in knowledge dimension was estimated from the principal investigators' publication counts and h-index. Data on research capacity-building were obtained from the Ministry of Health's information system. The informing decision-making dimension was analyzed from citations in Stork Network (Rede Cegonha) documents.

**RESULTS:**

Between 2002 and 2010, R$ 21.6 million were invested in 128 maternal mortality/morbidity projects. Over this period, the principal investigators published 174 articles, resulting in an h-index of 35, thus showing progress in the advancing in knowledge dimension. Within the research capacity-building dimension, training of 71 students (undergraduate/postgraduate) was observed. Progress in the informing decision-making dimension was modest: 73.5% of the 117 citations in the Stork Network documents were institutional documents and norms. One of the projects funded, the 2006/7 National Demography and Health Survey, was cited in program documents.

**CONCLUSION:**

Impacts were shown in the advancing in knowledge and research capacity-building dimensions. The health research system needs to incorporate research for evidence-informed policies.

## RESUMO

**CONTEXTO E OBJETIVO:**

Sistemas nacionais de pesquisa em saúde buscam gerar conhecimentos de qualidade para manter e promover a saúde da população. Este estudo visou analisar o impacto das pesquisas sobre morbimortalidade materna financiadas pelo Ministério de Saúde do Brasil e instituições parceiras, nas dimensões: avanços no conhecimento, construção de capacidade de pesquisa e tomada de decisão informada, da matriz da Canadian Academy of Health Sciences.

**DESENHO DO ESTUDO E LOCAL:**

Estudo descritivo baseado em dados secundários, realizado em universidade pública.

**MÉTODOS:**

A dimensão avanços no conhecimento foi estimada pelas publicações dos coordenadores de pesquisa e índice h. Dados sobre a capacidade de pesquisa foram obtidos no sistema de informação do Ministério da Saúde. A dimensão tomada de decisão informada foi analisada pelas citações nos documentos da Rede Cegonha.

**RESULTADOS:**

Foram investidos R$ 21,6 milhões de reais em 128 pesquisas sobre morbimortalidade materna entre 2002 e 2010. Nesse período, os coordenadores das pesquisas publicaram 174 artigos, resultando no índice h de 35, mostrando progressos na dimensão avanços no conhecimento. Na dimensão capacidade de pesquisa, foi constatado o treinamento de 71 estudantes (graduação e pós-graduação). Na dimensão tomada de decisão informada, o progresso foi modesto: 73,5% das 117 citações nos documentos da Rede Cegonha eram documentos institucionais e normas. Um dos projetos financiados, Pesquisa Nacional de Demografia e Saúde 2006/7, foi citado em documentos programáticos.

**CONCLUSÃO:**

Impactos foram demonstrados nas dimensões avanços no conhecimento e capacidade de pesquisa. O sistema de pesquisa em saúde necessita da incorporação de pesquisas para políticas informadas por evidências.

## INTRODUCTION

The national health research system has progressively strengthened in Brazil, mainly due to significant government funding of research projects and programs, along with human and institutional capacity-building. The increase in Brazilian scientific production is one of the advances resulting from these investments.^1^^,^^2^ In 2003, the process of setting the health research priorities agenda started with involvement of different social players (researchers; healthcare sector, education and science and technology decision-makers; healthcare system users; healthcare worker organizations; professional associations; and representatives of private companies).^3^^,^^4^ In 2004, the agenda^5^^,^^6^ was implemented in accordance with the recently published "good practice principles":^7^ inclusive process, information gathering, careful planning and funding policy, transparency and evaluation. The efforts that were made to guide health research policy have achieved and legitimated an unprecedented developmental upsurge in support for strategic health research.^4^

However, some components of the health research system in Brazil, such as use of research results and evaluation of their impact, are poorly structured and developed both by public institutions and agencies and by researchers themselves.^8^ Several authors have pointed out that the participatory process in setting research priorities contributes towards identifying the complex needs of the health system and services, and fosters an environment of opportunities for further research built on existing knowledge.^9^^,^^10^^,^^11^^,^^12^^,^^13^ Thus, on the one hand, this can improve both researchers' ability to provide answers to assist with understanding health determinants and their effects on health equity, and also their ability to develop new and better interventions for preventing and treating diseases in different population groups and social and political contexts. On the other hand, these scholars have pointed out the importance of effective management of agencies and institutions with regard to planning, promotion and implementation of funding mechanisms for efficient and equitable allocation of investment in research programs. According to these authors, the participatory process is one element that favors use and ownership of research results by the social players, with a potential impact on healthcare practices and policies within the healthcare sector and on strengthening the sector's own production and scientific capacity.

Over the past three decades, infant mortality rates have reduced substantially in Brazil, decreasing by 5.5% per annum in the 1980s and 1990s, and 4.4% per annum since 2000, to reach 14.6 deaths per 1000 live births in 2012; neonatal deaths account for 70% of infant deaths. On the other hand, official statistics show that maternal mortality ratios have remained stable over the past 10 years. Substantial challenges remain, including overmedicalization of childbirth (nearly 50% of babies are delivered by means of caesarean section), maternal deaths caused by illegal abortions and high frequency of preterm deliveries.^14^^,^^15^

In 2011, the Ministry of Health established the Rede Cegonha (Stork Network), with the aim of expanding access and improving the quality of prenatal care and assistance during delivery, postpartum care and child care for up to 24 months after birth.^16^ The initiative was based on the following laws: Law No. 8,080 dated November 19, 1990, which provides for the conditions for promotion, protection and recovery of health within the public healthcare services (SUS); Law No. 11,108 dated April 7, 2005, which guarantees mothers' right to have a companion with them during labor and the immediate postpartum period in SUS hospitals; and Law No. 11,634 dated December 27, 2007, which provides for the right of pregnant women to be linked to the maternity ward, where they will receive care under SUS. Rede Cegonha is a government priority.

The analysis on the impact of research on maternal mortality funded by the Ministry of Health and institutional partners may contribute towards strengthening institutions and funding agencies regarding the understanding of research processes, and towards identifying opportunities, difficulties and challenges relating to achieving the desired impacts from funded research.

## OBJECTIVE

This study focuses on the impact of research on maternal mortality funded by the Ministry of Health and institutional partners between 2002 and 2010, as measured by a series of well-established impact indicators.

## METHODS

### Analytical framework

The framework of the Canadian Academy of Health Sciences (CAHS) has five dimensions that make it possible to identify and evaluate the impact of research.^17^ This study focused on three of these: 1) advancing in knowledge; 2) research capacity-building; and 3) informing decision-making. The fourth dimension is health benefits, which recognizes progress regarding prevention, diagnosis, treatment, palliative care and progress in relation to health status, social and environmental risk factors, health determinants and changes to healthcare system performance. This, and the fifth dimension, economic and social benefits, were not included in the analysis since the data available did not cover the complex indicators of these two dimensions.

The advancing in knowledge dimension reveals new health research discoveries and progress, as well as contributions made to the scientific literature. The categories and indicators for this dimension were: (1) research activity: publication counts, i.e. the number and percentage of scientific publications and type; and (2) research quality: number of peer-reviewed journal articles, percentage of articles published in indexed journals, impact factor (h-index, is a measurement that aims to describe the scientific productivity and impact of a researcher or journal), and citations received per article (expressed as medians and interquartile intervals).

The research capacity-building dimension comprises development/enhancement of individual and team research skills in capacity-building for advances in knowledge. The category and indicator was research capacities, skills and personnel: number and percentage of students trained.

The informing decision-making dimension shows how research expands its outcomes and influences evidence-based policies. The categories and indicators were: (1) health-related decision-making: healthcare protocols, guidelines, manuals and technical norms, and technical methods of clinical evaluation; and (2) qualitative indicators: citations in public health policies and programs. The dimensions, categories and indicators were chosen for this study taking the following criteria into consideration: access and availability of data, data validity, reproducibility, cost and relevance.

### Data gathering and categories analyzed

Data on research investments over the period 2002-2010 were obtained from the Ministry of Health science and technology managerial information system <http://pesquisasaude.saude.gov.br/bdgdecit/> (accessed on December 10, 2015). For each project included in this study, identification data was collected (name of principal investigator (PI), title, summary, amount invested, year of contract, state/province and region), research capacity-building (number of undergraduate and postgraduate students involved; undergraduate course final dissertations, masters' dissertations and PhD theses concluded) and applicability of research results according to the PI.

Projects were selected using the filter "Women's Health" as the primary or secondary research agenda; this classification of projects into agendas is done by the PI at the time of project submission. The subgroup of projects relating to maternal mortality and morbidity and the type of research approach were classified by two of the authors in a blind and independent manner, based on project titles and summaries; in four cases the summary was missing and those projects were classified based only on the title (none of them were related to maternal mortality).

The type of research approach was classified according to the definition used by the Canadian Academy of Health Sciences (CAHS).^17^ CAHS has defined that health services research (HSR) examines how people get access to healthcare, how much care costs and what happens to patients as a result of this care. The main goals of HSR are to identify the most effective ways to organize, manage, finance and deliver high-quality care; reduce medical errors; and improve patient safety.^18^ Population and public health research (PPHR) is defined as research with the goal of improving population health, or of defined subpopulations, through better understanding of the ways in which social, cultural, environmental, occupational and economic factors determine health status. The ambit of PPHR includes research into the complex interactions (biological, environmental, social and cultural) that determine the health of individuals, communities and global populations.^19^

Clinical research (CR) has "the goal of improving the diagnosis and treatment (including rehabilitation and palliation) of disease and injury; improving health and quality of life of individuals as they pass through normal life stages". Biomedical research (BR) is conducted "with the goal of understanding normal and abnormal human functioning at the molecular, cellular, organ system and whole body levels, including development of tools and techniques to be applied for this purpose; developing new therapies or devices that improve health or the quality of life of individuals, up to the point where they are tested on human subjects".^17^

One indicator for the advancing in knowledge dimension was "publication counts", i.e., the number and percentage of scientific publications according to the type of research approach. We accessed the list of articles on maternal mortality and morbidity included in the curricula of the PIs funded by the Ministry of Health and partners. All articles published from 2002 to 2010 and informed in the curricula were extracted from the Lattes Platform, which is the major platform for curricula encompassing virtually every Brazilian researcher, using the ScriptLattes software (http://buscatextual.cnpq.br/buscatextual/busca.do). The data were exported to a RIS (Research Information Systems) file and then imported to Mendeley Desktop; duplicated articles were excluded, thus resulting in a total of 2,961 articles. These were then classified according to whether they related to maternal mortality and morbidity, based on the list of selected keywords appearing in the article titles. This resulted in identifying 174 articles on maternal mortality and morbidity. In addition, the number of dissertations and theses developed during the project was calculated according to information provided by the principal investigator in the Ministry of Health S&T information system <http://pesquisasaude.saude.gov.br/bdgdecit/>.

The research quality category was assessed according to the number of peer-reviewed journal articles, percentage of articles published in indexed journals, impact factor (h-index) and citations received per article (expressed as medians and interquartile intervals). The Google Scholar database (http://scholar.google.com) was used to obtain the number of citations received by each article, and journals were considered to be indexed when included in Google Scholar metrics. The h-index was calculated based on Google Scholar citations.

With regard to the research capacity-building dimension, the number of undergraduate and postgraduate students trained through the projects was calculated according to the information provided by the principal investigator in the abovementioned Ministry of Health science and technology information system.

With regard to the informing decision-making dimension, one of the focus area was Rede Cegonha*,* the government initiative that was launched in 2011 to reduce maternal mortality and morbidity. We analyzed clinical protocols, guidelines and manuals published by the Ministry of Health, which could indicate the use of evidence produced by these projects in the health-related decision-making category.^20^^,^^21^^,^^22^^,^^23^^,^^24^ Citation in public health policy documents was also considered, since we investigated Rede Cegonha policy documents.^25^^,^^26^^,^^27^ We also investigated what the principal investigators pointed out as forms of applicability of research results in the category "health-related decision-making", which they registered in the Ministry of Health S&T information system.

These publications were classified as "grey literature", in accordance with the United Kingdom Department of Health definition: "produced at all levels of government, academia, business and industry in print and electronic formats, but which is not controlled by commercial publishers; it may also be defined, broadly, to include everything except peer-reviewed books and journals".^28^

## RESULTS

Between 2002 and 2010, the Ministry of Health and partner institutions funded 128 research projects on maternal mortality and morbidity (Table 1). The distribution of maternal mortality and morbidity projects per year showed that 78 proposals (56% of the total) were approved in 2004 and 2007, when two large Calls for Proposals were launched: "Maternal Mortality and Neonatal Morbidity and Mortality"^29^ in 2004, and "Women's Health"^30^ in 2007.

**Table 1:**
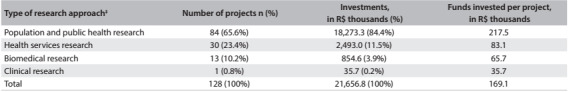
Research projects on maternal mortality and morbidity funded by the Ministry of Health and its partners, according to type of research approach. Brazil 2002-2010*,†

*Partner institutions: Conselho Nacional de Desenvolvimento Científico e Tecnológico (CNPq), Ministério da Ciência e Tecnologia (MCT), Financiadora de Estudos e Projetos (Finep), Fundações Estaduais de Amparo à Pesquisa (FAPs); †Data captured on December 10, 2015; ‡According to the Canadian Academy for Health Sciences.

Regarding the approach, there were 84 projects on Population and Public Health Research (PPHR), which received by far the largest investment (Table 1). The investment per project was R$ 217,000 and R$ 83,000 in the first and second project approach, respectively. On average, HSR projects received less than half the investment made in PPHR.

**Figure 1:**
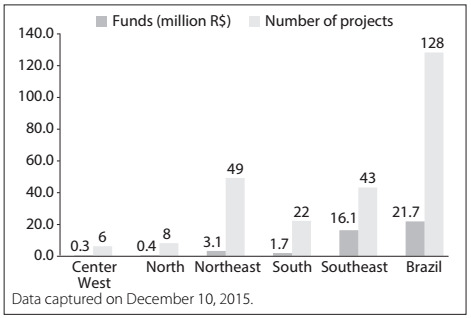
Investments and numbers of projects within maternal mortality and morbidity research funded by the Ministry of Health and partner institutions, according to geographical regions. Brazil, 2002-2010.

With regard to regional distribution, the institutions receiving funding (mostly public universities and public research institutes) were predominantly located in the northeastern region (38%) and southeastern region (33%). However, in analyzing the amount of investments, the southeast received five times more resources than the northeast (Figure 1).

Analysis of the advancing in knowledge dimension according to publication counts found 245 publications: 174 scientific articles (Table 2) and 71 other types of scientific publications: doctoral theses (16), master's dissertations (41) and undergraduate end-of-course dissertations (14). Articles with a Population and Public Health Research approach predominated. Regarding postgraduate products, the majority of the publications were master's dissertations. Only one patent was deposited.

**Table 2:**
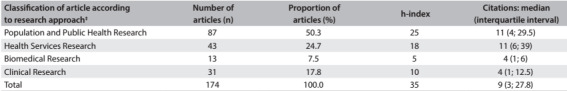
Articles published on maternal mortality and morbidity that were included in the curricula of researchers funded by the Brazilian Ministry of Health and its partners, according to type of research approach*,†. Brazil, 2002-2010

*Partner institutions: Conselho Nacional de Desenvolvimento Científico e Tecnológico (CNPq), Ministério da Ciência e Tecnologia (MCT), Financiadora de Estudos e Projetos (Finep), Fundações Estaduais de Amparo à Pesquisa (FAPs); †Data captured in June 2014; ‡According to the Canadian Academy for Health Sciences.

With regard to research quality, the 174 papers produced had an h-index of 35, and 154 (89%) were published in indexed journals. The median number of citations received by each paper was 9, with an interquartile interval from 3 to 27.75. Articles classified as Population and Public Health Research or as Health Services Research had a higher impact than those in the other two categories (Table 2). Table 3 ^31^^,^^32^^,^^33^^,^^34^^,^^35^^,^^36^^,^^37^^,^^38^^,^^39^^,^^40^^,^^41^^,^^42^ presents the top three most cited articles for each type of research approach. Five out of the 12 examples resulted from research conducted abroad; clear predominance of international cooperation was found among biomedical and clinical research. However, most of these research results are applicable to all pregnancies, and therefore can be extrapolated to a Brazilian scenario.

**Table 3:** Top three most cited articles published on maternal mortality and morbidity by investigators funded by the Brazilian Ministry of Health and its partners, according to type of research approach. Brazil, 2002-2010

Research approach* and article title	Year	Journal reference	Citations
Population and public health research			
Maternal mortality in Brazilian State Capitals: some characteristics and estimates for and adjustment factor	2004	Rev Bras Epidemiol, 7(4):449-60.^31^	187
Racial, sociodemographic, and prenatal and childbirth care inequalities in Brazil, 1999-2001	2005	Rev Saude Publica, 39(1):100-7.^32^	151
Cesarean sections: who wants them and under what circumstances?	2003	Cad Saude Publica, 19(6):1611-20.^33^	98
Health services research			
Aspects of women's satisfaction with childbirth care in a maternity hospital in Rio de Janeiro	2004	Cad Saude Publica, 20(Sup 1):S52-S62.^34^	124
Quality of birth care in maternity hospitals of Rio de Janeiro, Brazil	2005	Rev Saude Publica, 39(4):646-54.^35^	102
Adequacy of the two references services for women with high risk pregnancies at maternity hospitals of the Brazilian Public Health System in the city of Recife, in the State of Pernambuco	2007	Rev Bras Saúde Matern Infant, 7(3):309-17.^36^	81
Biomedical research			
The pregnancy-induced increase of plasma angiotensin-(1-7) is blunted in gestational diabetes	2007	Regul Pept, 141(1):55-60.^37^	25
Increased serum phosphodiesterase activity in women with pre-eclampsia	2006	Br J Obstet Gynaecol, 113(5):577-9.^38^	17
Increased circulating thrombomodulin levels in pre-eclampsia	2008	Clin Chim Acta, 387(1-2):168-71.^39^	12
Clinical research			
Do women with pre-eclampsia, and their babies, benefit from magnesium sulphate? The Magpie Trial: a randomized, placebo-controlled trial	2002	Lancet, 359(9321):1877-90.^40^	475
Comparison of Magnesium Sulfate and Nimodipine for the Prevention of Eclampsia	2003	N Engl J Med, 348(4):304-11.^41^	233
Beneficial interventions for maternal mortality prevention in the prenatal period	2006	Rev Bras Ginecol Obstet, 28(5):310-5.^42^	71

*According to the Canadian Academy for Health Sciences.

The research capacity-building dimension was evaluated according to information provided by the principal investigators: 71 undergraduate and postgraduate students trained during the different stages of development of the research.

The informing decision-making dimension, focusing on the use made of research results in official protocols, guidelines, manuals and healthcare policy documents published through the recently launched program to reduce maternal mortality (Rede Cegonha), showed poor use made (Table 4). The eight official Brazilian Ministry of Health documents were analyzed and 117 citations were compiled. The vast majority (73.5%) could be classified as grey literature. The Demography and Health Survey, which was one of the projects analyzed here, was cited in two Ministry of Health documents.

**Table 4:**
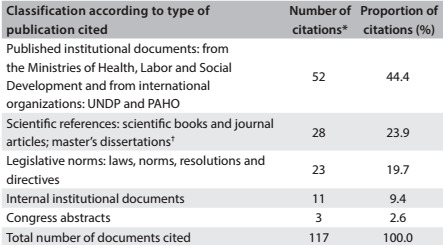
Citation of scientific evidence in the official protocols, guidelines, manuals and health policy documents published by the Ministry of Health for Rede Cegonha ("Stork Network"). Brazil 2011-2013*

*All the eight official Rede Cegonha documents were analyzed, totaling 117 citations; †18 books published in Brazil, 5 books published abroad, three articles published in Brazil; two master's dissertations. UNDP = United Nations Development Programme; PAHO = Pan-American Health Organization.

Sixty-eight researchers (53%) pointed out various forms of applicability of research results in the category health-related decision-making. Table 5 presents a synthesis of the reported applications. Knowledge of risk factors, especially in relation to infectious and hypertensive disorders during pregnancy, formulation of policies and implementation of strategies and actions for improving maternal and newborn health, prevailed as potential evidence of use of research results. Healthcare service managers and healthcare workers were cited as potential users of research results. However, few changes to healthcare services were cited by the principal investigators, regarding the real use of the evidence produced.

**Table 5:**
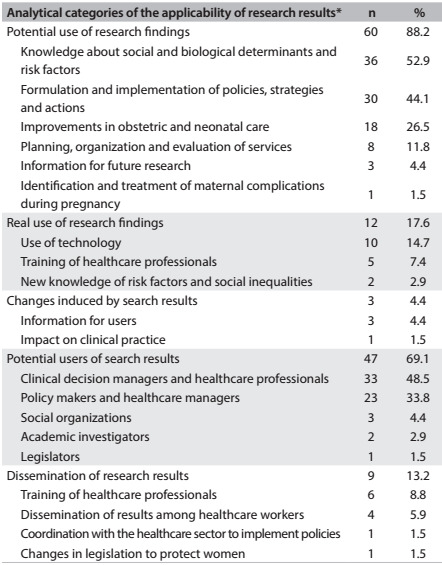
Perspective of the principal investigators of research projects financed by the Ministry of Health and partners, regarding the applicability of research results within the informed decision-making dimension. Brazil, 2002-2010

*Multiple answers allowed.

## DISCUSSION

The aim of this study was to analyze the contributions made by the investments and the incorporation of research findings regarding maternal mortality and morbidity in public health policy formulation in Brazil. This analysis was conducted based on three analytical dimensions of the CAHS framework, with emphasis on citations of articles, dissertations and theses in preparing the official Rede Cegonha public policy documents*.* This public policy was formulated in the form of a care network "to ensure women's right to reproductive planning and humanization of the pregnancy, childbirth and postpartum periods, as well as the child's right to safe birth and healthy growth and development".^16^

The predominance of institutional documents and legislative norms among the citations of official publications reflects policymakers' practice of prioritizing existing technical and legislative regulations to guide health managers primarily with regard to organization, functioning and physical restructuring of the healthcare network, including intensive care units, provision of obstetric and neonatal care and transfer of funds. The scarce use of scientific references reaffirms the difficulty in incorporating knowledge to promote improvements in healthcare systems that have been pointed out in several studies.^43^^,^^44^^,^^45^^,^^46^ However, it also reveals the challenge of measuring the influence of research findings on decision-making, because of the inherent dynamics of the political process in healthcare management.^47^^,^^48^

At the same time, this poses questions regarding the ability of researchers to provide answers that will enable interventions that promote significant changes towards safe motherhood, improve professionals' technical quality and clinical practices and enhance system performance and healthcare programs.^45^^,^^46^

However, in order for researchers to meet this demand, supportive environments are needed, so that on the one hand, researchers can be brought closer to the needs of healthcare services and, on the other hand, substantial investment in HSR can be made by public institutions and agencies, thereby promoting the participation of healthcare institutions.^13^^,^^17^ As pointed out earlier, although HSR accounted for 23.0% of the projects funded, this represented only 11.5% of the total amount of investments.

Strengthening of the national health research system requires efforts to understand the needs of the players in the system and, in particular, strategies to promote translation and communication of research results, so as to inform policy and healthcare practices. The traditional forms of dissemination of research results among scientists are not adequate, and innovative ways are needed in order to foster access to the results and recommendations for the healthcare system.

Another point to be highlighted concerns the distribution of research funds in Brazil. In general, larger investments are targeted to areas with the best science and technology capabilities. As our results demonstrated, the southeastern region, which has better-structured science and technology capacities, received five times more resources, despite the fact that there were more projects in the northeastern region. However, the Brazilian health research system needs to achieve a balance between the distribution of funds according to science and technology capacity, while also taking into account local health situations and priorities. In the case of maternal mortality, there are profound regional, ethnic and socioeconomic disparities: higher maternal mortality levels affect the population in the poorer northern and northeastern regions, compared with the more affluent southern and southeastern regions.^15^ Research investments could promote equity through projects to investigate better access to healthcare, organization of care processes and ways of improving clinical practice and women's safety during the prenatal, childbirth and postpartum periods.^14^^,^^49^ These subjects could be studied by research institutions in the regions most affected, through networking with the more traditional and consolidated science and technology institutions.

There is some evidence that investigators in the northeastern region respond very well to research funds allocated there. The scientific publications resulting from the Research for SUS Program (PPSUS) over the period 2004-2009 comprised 1,020 master's dissertations and doctoral theses. The northeastern region reported the largest number of academic degrees: 322 master's and doctoral degrees (32%), i.e. ahead of the more developed and populated southeastern region.^4^

The Brazilian health research system therefore needs to strengthen research functions, such as monitoring of funding distribution mechanisms, development of human and physical capacity to conduct, absorb and use research, and evaluation of the impacts of research. One noteworthy issue is the challenge of promoting a culture that encourages involvement of investigators in policy and decision-making while, on the other hand, facilitating interaction between researchers and policymakers, service managers, providers, healthcare workers and service users.^50^ One proposal is to increase investments and strategies for HSR through participation of healthcare service managers, workers and users in formulating and implementing research and incorporating its results.

The EVIPNet Brazil initiative can be highlighted. This was started in 2009 and is run by the Ministry of Health and the Pan-American Health Organization (PAHO). Its objective is to establish mechanisms to facilitate the use of relevant scientific evidence in formulating and implementing health policies (http://brasil.evipnet.org/).

The investigators funded by the Ministry of Health and partners produced a reasonable number of high-quality papers, as showed by an h-index of 35 and the observation that nearly 90% of the articles were published in indexed journals. This is an indication that research projects on maternal mortality and morbidity generated important scientific results and can be used as an important means of measuring funding efficiency. Articles classified as Population and Public Health Research (h-index of 25) or as Health Services Research (h-index of 18) had higher impacts, with higher medians for citations received and also a much higher top quartile. We can infer that Brazilian researchers who developed Population and Public Health Research relating to maternal mortality and morbidity tended to produce articles with higher citation indexes than those dedicated to the Biomedical field. However, Clinical Research accounted for the top two most cited articles.

There are some limitations to consider in relation to using article output as a funding success indicator. Some research projects may result in articles published years later, and therefore not included in our analysis. It is hard to determine a reasonable time interval for funded research to generate publications. Nonetheless, it should be noted that research that is more complex or takes longer to conduct might generate published papers with higher impact on a future date. This is primarily because different types of research will have different timetables: an *in vitro* study will most likely produce faster results and publication than a cohort study or health services research. Moreover, some research may gather additional funding to expand its objectives, methodology or sampling, thus delaying publication but also producing more relevant information. In this way, some papers receive support from more than one institution, while rarely mentioning the real contribution of each source of funding.

The publications list for each author was extracted from the Lattes Platform, which is an extremely reliable but self-informed mechanism, and which might result in underreporting to some extent. The classification of research approach using only the title was a limiting factor. Despite those limitations, the number and quality of publications are considered to be good ways of measuring research impact.

With regard to the informing decision-making dimension, we used most of the indicators suggested by the Canadian Framework, such as citation of research results in clinical protocols, guidelines and manuals published by the Ministry of Health for policy, programs or healthcare. However other indicators could be used, such as citation of research results for capacity-building and training of healthcare professionals, and in the media (newspaper articles and interviews in the press); this constitutes a study limitation.

## CONCLUSION

Impacts were shown with regard to the advancing in knowledge and research capacity-building dimensions. However, the Brazilian national health research system needs strengthened mechanisms for informing evidence-based policies and promoting incorporation and use of research results into policy formulation, strategies and actions, in order to improve maternal and newborn healthcare services. Recognition of the relevance of health research investments is strengthened insofar as it can be shown to produce impacts on policy, and this requires engagement of researchers for effective interaction with various sectors of society, and in particular healthcare policy stakeholders.
